# The complete mitochondrial genome and phylogenetic analysis of *Upogebia major* (De Haan, 1841)

**DOI:** 10.1080/23802359.2020.1870898

**Published:** 2021-03-18

**Authors:** Xiaoyue Sun, Jun He

**Affiliations:** College of Life Sciences, Yantai University, Yantai, China

**Keywords:** *Upogebia major*, mitochondrial genome, phylogenetic tree analysis

## Abstract

The complete mitochondrial (mt) genome of *Upogebia major* (De Haan, 1841) is 16,131 bp in length, comprising 13 protein-coding genes (PCGs), 22 transfer RNA genes, and two ribosomal RNA genes. The nucleotide composition for *U. major* is 34.3% of A, 36.6% of T, 10.9% of C, and 18.3% of G. All PCGs are initiated by ATN codons, except for the *cox*1 gene, which was not determined. Nine PCGs use a common stop codon of TAA or TAG, the other four end with an incomplete stop codon (a single stop nucleotide T). Phylogenetic tree analysis showed that *U. major* had a close relationship with the species from the family Thalassinidae. The study will provide an important theoretical basis for further analysis of mt genome evolution and the phylogenetic relationships of the order Decapoda.

*Upogebia major* (De Haan, 1841) (Crustacea, Decapoda, Gebiidea) is one of the largest upogebiids, which distributes widely in coastal shallow waters of the Shandong Peninsula, the Japanese Islands, and the Korean Peninsula, and lives in burrows that extend deep into the sediment (Felder 2001; Kinoshita et al. 2008; Selin 2017). *U. major* is an active edificator and defines the abundance and composition of the intertidal fauna (Selin 2017). Based on the abundance and bioturbation, *U. major* plays an important role in the structure of benthic community that arouses more and more attention (Dworschak 2000).

The total genome DNA was extracted from the samples that were collected from Yantai, Shandong province (37°28′N, 121°37′E). The samples were stored in 100% ethanol immediately after collection and then preserved at −80 °C (Pu et al. 2017). The specimen was deposited in the marine specimen room of Yantai University with an accession number YTU-SXY-201900370003. The mt genome was sequenced using Illumina NovaSeq 6000 sequencing platform (Illumina, San Diego, CA) and assembled with the programs SPAdes v3.10.1 (http://bioinf.spbau.ru/spades). Generous Prime was used for gene statistics and MrBayes 3.2 program (Ronquist et al. 2012) was used for phylogenetic tree construction. The complete mt genome was annotated using MITOS web server (http://mitos.bioinf.uni-leipzig.de/index.py). All work was conducted with the formal approval of the local human subject or animal care committees (institutional and national), and that clinical trials have been registered as the Guidelines of Animal Ethics published by the National Institute of Biological Resources (NIBR).

The complete mitochondrial (mt) genome of *U. major* was 16,131 bp in length (GenBank accession number: MT762288). The overall base composition is A = 5526 bp (34.3%), T = 5898 bp (36.6%), C = 1759 bp (10.9%), and G = 2948 bp (18.3%), with a GC content of 29.2%. Similar to the mt genomes of other Crustacea, the GC content is lower than the AT content (Tsang et al. 2015; Song et al. 2020). The genome contains 13 protein-coding genes (PCGs), including seven NADH dehydrogenase (*nad*1, *nad*2, *nad*3, *nad*4, *nad*4L, *nad*5, and *nad*6), three cytochrome c oxidase (*cox*1, *cox*2, and *cox*3), one cytochrome b (cytb), and two ATP synthase (*atp*6 and *atp*8). Meanwhile, the genome contains 22 tRNA genes and two rRNA genes. The total length of 13 PCGs is 11,161 bp. Nine of the 13 PCGs start with ATG (*nad*1, *nad*4, *nad*4L, *nad*5, *cox*2, *cox*3, *cytb*, *atp*6, and *atp*8), and three start with ATT (*nad*2, *nad*3, and *nad*6), whereas the initiation codon of *cox*1 could not be determined. This phenomenon is also found in other shrimps, such as *Rhynchocinetes durbanensis* (Tang et al. 2016). Nine of the 13 PCGs using stop codons TAA (*nad*2, *nad*3, *nad*4L, *nad*6, *atp*6, *atp*8, and *cox*1), and TAG (*nad*1, *nad*4). It is worth noting that the remaining four PCGs terminate with an incomplete stop codon T (*cox*2, *cox*3, *nad*5, and *cytb*), which is completed by the addition of 3′ A residues to the mRNA. There are two ribosomal RNA genes, including *12SrRNA* (833 bp) and *16SrRNA* (1381 bp), which are commonly isolated by a *tRNA-Val* (GTA). All tRNAs have the typical cloverleaf structure ranged from 63 to 71 bp in length (Lowe and Chan 2016). The gene arrangement and transcriptional polarity are partially different from that of the Japanese ghost shrimp *Nihonotrypaea japonica*, which belongs to the infraorder Axiidea (Kim et al. 2013). Gene order and content are identical in *Upogebia yokoyai* (Lin et al. 2012) and *Upogebia pusilla* (Shen et al. 2013) mt genomes, except for *trn*L, which is unique in the mt genome of *U. major* (Yang et al. 2016). Since there are little data about the complete mt genome sequences in the infraorder Upogebiidae yet, this report will provide additional information in relation to mt genome diversity and the evolution of the decapods.

In order to better elucidate the phylogenetic relationship between *U. major* and its 11 reported close relative species, the mt genome sequences of these species, as well as *Squilla mantis* (GenBank accession number: NC_006018) served as outgroup, were downloaded from the GenBank database. Thirteen PCGs from 13 mt genome DNA sequences were used to construct phylogenetic tree by MrBayes 3.2 with the Bayesian inference (BI) method. Support values for each node were calculated from Bayesian posterior probability (BPP). The result tree confirmed that *U. major* was clustered with *U. yokoyai* and rooted with the other Upogebiidae species (Figure 1). The conclusion drawn from this phylogenetic tree was that Upogebiidae was relatively distant from Calliannassidae which belonged to the order Axiidea, and the family of Thalassinidae and Upogebiidae were the closest, both of which belonged to the order Gebiidea, which have not been previously reported. These results provide insights into crustacean mt genome diversity and evolution of the decapods.

**Figure 1.**
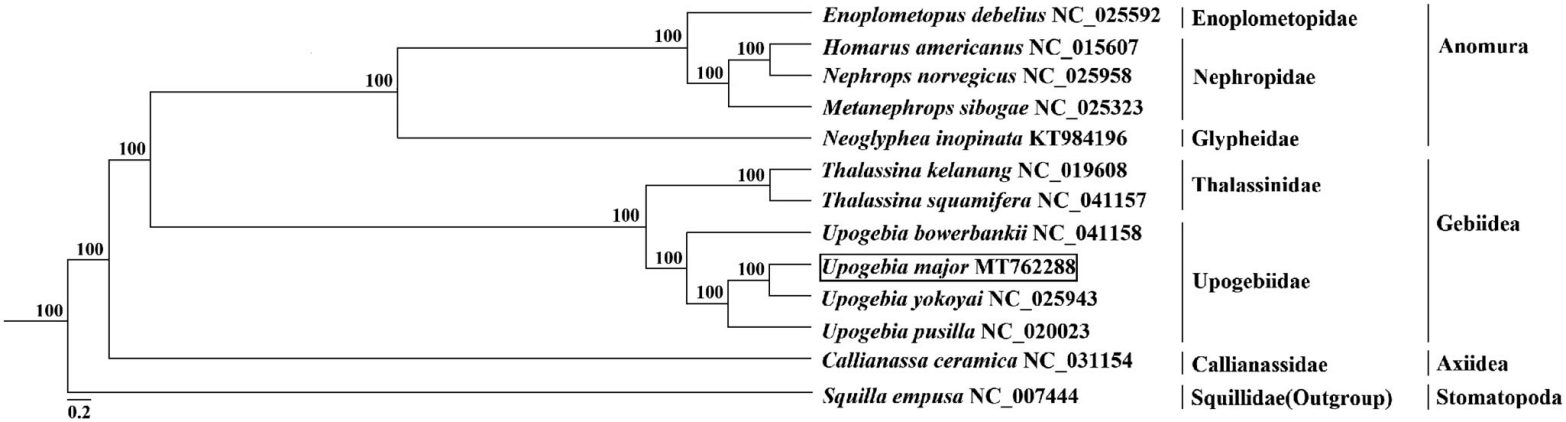
Phylogenetic position of *U. major* based on mt genome sequences. Twelve in-group (Decapoda) and one out-group (Stomatopoda), respectively. The numbers within brackets after the species show the accession numbers of used mt genome sequences from GenBank.

## Data Availability

The data that support the findings of this study are openly available in GenBank at https://www.ncbi.nlm.nih.gov, GenBank accession number: MT762288.
